# Notes in the Practice of

**Published:** 1888-07

**Authors:** G. G. Roy

**Affiliations:** Atlanta, Georgia; Professor of Materia Medica in Southern Medical College


### Southern Medical Record.

A MONTHLY JOURNAL OF PRACTICAL MEDICINE.

☞All Communications must be addressed to R. C. Word, M. D., Managing Editor.

"*Quicquid Præcipies Esto Brevis.*"

Vol. XVIII. ATLANTA, GA., JULY, 1888. No. 7.

### ORIGINAL AND SELECTED ARTICLES.

### NOTES OF CASES IN THE PRACTICE OF

G. G. Roy, M. D., Atlanta, Georgia.

Professor of Materia Medica in Southern Medical College.

On the 29th day of December, 1887, Lou Jessee Alford. white female, twelve years of age, in attempting to recross the Georgia Pacific railroad track in this city had her right foot and ankle so badly crushed (the engine which was attached to the train being put in motion before she cleared the track—the engineer nor any of the train hands having any knowledge of the presence of any person on or about the track) as to require amputation. This was done with the assistance of Drs. J. McFadden Gaston, N. O. Harris, and my son, C. D. Roy, a medical student.

The location of the hut where the child lived, and her surroundings, were very unfavorable for the successful treatment of such an injury. She had sloughing of the flaps and erysipelas of the limb, and necessarily became very weak. These complications over, the stump assumed healthy granulation, and without the accident to be mentioned she was in a fair way to recovery.

From the date of the operation it had been necessary to give her, at the daily dressing of the wound, a small quantity of an anæsthetic, the a. c. e. formula (alcohol, chloroform and ether), just sufficient to produce insensibility to pain. On the 28th day of February, 1888, a much smaller portion than usual, it being all on hand, was poured upon a handkerchief, and put to the nose by the child herself, and watched by her aunt, who was employed by me to nurse her, and who had been using it in this way for nearly two months at each daily dressing of the limb. I began to remove the dressing, but in not more than a minute, looked up and noticed that she was looking unusually pale, and breathing irregularly, detected no pulse at the wrist, and the faintest heart-beat. I began immediately artificial respiration, keeping the head low, hypodermic injections of ether, whiskey and spts. ammo., besides using all the stimulating rubifacent means at my command, but without avail, she did not revive at all, and was dead in five minutes.

Now, what could have occasioned this sudden and unexpected paralysis of the heart?

A larger quantity of the "a. c. e." mixture had been used every day for two months to benumb the sensibility to pain when dressing the injury. She seemed as bright and in as good general health that morning as she had been since the accident. The anæsthetic was administered in the same way and by the same party under my supervision, except that barely one-third of the usual quantity was used on this occasion, for the reason before mentioned.

There was no indication whatever, pointing as a warning, that anything was wrong with the child. Pulse, respiration and general condition apparently as good that morning as usual.

I am free to admit that such an accident in my hands, was the severest mental shock of my life, and would not experience another such for all the wealth of this world.

The only solution, at all plausible to my mind, is this, (and I do not recollect of seeing any warning of it in the literature of anæsthetics) that the daily use of the anæsthetic for so long a period, had gradually weakened the heart, until on the fatal occasion, it had arrived at the point when it could not stand longer a like strain, and as a flash of lightning from a passing cloud may, without warning, paralyze or even destroy the human body, so this was done. The weaker succumbs to the stronger force.

I hope these notes may elicit the views of the intelligent readers of the RECORD on this case.

### A CASE OF PERINEPHRETIC ABSCESS IN A LADY SIXTY YEARS OF AGE.

I was called to Mrs. T. G., a lady whose health had been poor for the last ten years, but being of industrious habits and strong will-force rarely yielded to her ill-feelings to the extent of either "taking her bed or room." On the 15th day of November, 1887, I found her with an aggravated attack of (to her not unusual) liver and dyspeptic trouble. This, in a few days, yielded to ordinary treatment; but then a new complication presented itself in the form of what appeared to be an onset of muscular rheumatism involving the left leg and the lumbar muscles of the same side, attended with marked exacerbations of fever. Under proper treatment the rheumatic symptoms were greatly relieved in a few days, especially those of the limb, while the pain of the back and side were more aggravated and persistent, and had changed their rheumatic character. Stimulating and even pustulating rubefacients were used over seat of pain, and constitutional treatment was likewise resorted to, both giving only slight relief at intervals.

After three weeks confinement to bed the exacerbations of fever, preceded by chilly sensations, were more pronounced and were attended with the peculiar flush of the cheeks so indicative of serious structural disintegration.

About this time marked swelling of the left side in the region of the kidney, with great tenderness on pressure, indicating the formation of an abscess, was quite manifest.

Anodyne fomentations were applied, the supporting treatment reinforced by brandy, tr. iron, quinine and chlorate potassium, with occasional doses of opium to relieve pain and procure rest. Believing that deep seated fluctuation was detected just below the left kidney, but preferring to have my diagnosis confirmed, I invoked the assistance and judgment of my colleague, Dr. J. McF. Gaston, when an exploratory puncture with an aspirating needle was made, resulting in the discovery of pus, as I had anticipated. A deep incision was made with the bistouri through the lateral abdominal walls, which gave exit to a large quantity of healthy pus.

There was quite a large surface of indurated tissue surrounding the abscess and to give free outlet to this pus, from the breaking down of this, a drainage tube was inserted through which there was a free flow for several weeks. And even after the tube was removed from the discomfort it gave, there was a free discharge for five or six weeks longer, especially when pressure was made upwards from the direction of the sacro-iliac junction of that side, where it seemed to gravitate. From the beginning, the pus was laudable; only on one or two occasions appearing a little dark and grumous, with slightly unpleasant odor, when considerable pressure was used to dislodge it from a deep seated pocket.

The abscess continued to discharge for eight weeks, yielding only yellow serum in small quantity in the last two weeks of its existence.

Her general health has improved, an harassing cough which had troubled her for several years has about subsided, and at this writing she is going about the house, and apparently has a brighter prospect of good health than she has had for ten years.

A CASE OF PLACENTA PREVIA CENTRALIS—RECOVERY.

I was consulted by Mr. T. on January 22, 1888, in regard to his wife's condition, which appeared to him alarming, and he was unable to find the physician who had been attending her during the past forty-eight hours. He reported that his wife "was about her term," and that for the past forty-eight hours had been flooding at intervals so profusely that then she was so weak she could not raise her head from the pillow without fainting.

I gave him "an emergency ℞." and advised him to get his physician as soon as possible, as I apprehended from the symptoms and the history he had given, that a placenta previa was present, and told him that labors with that complication were dangerous, and the physician should be ready at hand for any emergency.

The ℞., f. e. ergot, f. e. blackhaw, f. e. witchhazel, with 6 gtts. elix. opi in each drachm, checked the hemorrhage for several hours. Early in the night he called again, stating that he had been still unsuccessful in finding his physician, and as there had been another recurrence of hemorrhage (though less profuse) he wished another recipe. I gave him the officinal pills of acetate lead and opium, and directed one pill every hour in the event of profuse hemorrhage untill he could get a physician.

Next morning, still not finding his physician, he asked me to visit and examine her.

The examination revealed a well defined placenta previa centralis, completely covering the os, which was patulous and flabby. Up to this time she had not had a labor pain, and the presenting part of the child could not be distinctly made out through the thickness of the placenta.

I applied over the os a pledget of absorbent cotton covered with a powder of equal parts of alum and tannin, and over this tamponed the vagina closely with absorbent cotton.

In the afternoon my friend Dr. Gaston visited the patient with me and after careful examination confirmed the diagnosis. I requested him to hold himself in readiness to promptly respond to a call if I was out of the way or might require his assistance, and appointed the hour of 8 o'clock, p. m., to return. This appointment I was able promptly to meet, and just before reaching the house met her husband on his way to summons me through the nearest telephone.

On reaching her bedside, I was informed that she had a "hard pain" and must be in labor, but there was no hemorrhage.

An examination was promptly made and a breech presentation discovered, the cone-shaped buttox of the child forming a wedge-shaped mass which firmly compressed the torn surface of the placenta and prevented further hemorrhage, just on the same principle that hemorrhages are arrested in these cases by turning in head presentations, and bringing down a foot, and by traction, making a tampon of the lower extremity as recommended by Braxton Hicks. The next pain brought the fœtus, which had evidently been dead a week or more.

Rapid delivery of the placenta was made, the womb contracted well, and in spite of the enfeebled condition of the woman from excessive hemorrhage before delivery, she made a rapid and excellent recovery.

